# Referent data for investigations of upper limb accelerometry: harmonized data from three cohorts of typically-developing children

**DOI:** 10.3389/fped.2024.1361757

**Published:** 2024-03-01

**Authors:** Catherine E. Lang, Catherine R. Hoyt, Jeffrey D. Konrad, Kayla R. Bell, Natasha Marrus, Marghuretta D. Bland, Keith R. Lohse, Allison E. Miller

**Affiliations:** ^1^ Program in Physical Therapy, Washington University School of Medicine, St. Louis, MO, United States; ^2^ Program in Occupational Therapy, Washington University School of Medicine, St. Louis, MO, United States; ^3^ Department of Neurology, Washington University School of Medicine, St. Louis, MO, United States; ^4^ Department of Psychiatry, Washington University School of Medicine, St. Louis, MO, United States

**Keywords:** wearable sensors, upper limb, movement, accelerometers, pediatrics, behavior, outcomes

## Abstract

**Aim:**

The rise of wearable sensing technology shows promise for addressing the challenges of measuring motor behavior in pediatric populations. The current pediatric wearable sensing literature is highly variable with respect to the number of sensors used, sensor placement, wearing time, and how data extracted from the sensors are analyzed. Many studies derive conceptually similar variables via different calculation methods, making it hard to compare across studies and clinical populations. In hopes of moving the field forward, this report provides referent upper limb wearable sensor data from accelerometers on 25 variables in typically-developing children, ages 3–17 years.

**Methods:**

This is a secondary analysis of data from three pediatric cohorts of children 3–17 years of age. Participants (*n* = 222) in the cohorts wore bilateral wrist accelerometers for 2–4 days for a total of 622 recording days. Accelerometer data were reprocessed to compute 25 variables that quantified upper limb movement duration, intensity, symmetry, and complexity. Analyses examined the influence of hand dominance, age, gender, reliability, day-to-day stability, and the relationships between variables.

**Results:**

The majority of variables were similar on the dominant and non-dominant sides, declined slightly with age, and were not different between boys and girls. ICC values were moderate to excellent. Variation within individuals across days generally ranged from 3% to 32%. A web-based R shiny object is available for data viewing.

**Interpretation:**

With the use of wearable movement sensors increasing rapidly, these data provide key, referent information for researchers as they design studies, and analyze and interpret data from neurodevelopmental and other pediatric clinical populations. These data may be of particularly high value for pediatric rare diseases.

## Introduction

Assessment of motor behavior in pediatrics is challenging. Research and clinical care often depend on standardized motor assessments administered by trained professionals (1). Standardized assessments, however, are time-consuming and assess behavior during a limited clinical or laboratory encounter. Questionnaires completed by a caregivers or teachers provide alternative assessment options that are quick and cheap, but may be less accurate and more subject to bias (2). Use of either standardized assessments or questionnaires to quantify enduring aspects of motor behavior can also be complicated by the need for a different version of an assessment or a completely new assessment tool designed to evaluate advancing motor skills during development. The rise of wearable sensing technology shows promise for addressing these challenges in assessment of motor behavior across the lifespan (3). Accelerometers in wearable devices worn on the upper limbs can quantify real-life motor behaviors, providing continuous monitoring over longer periods of time. Devices are small and can be readily worn by children of all ages. Extracted accelerometry data can then be used to compute numerous variables capturing a range of movement characteristics (3).

Beyond its use quantifying physical activity, accelerometry is emerging as a tool to measure upper limb motor behavior in in children with Cerebral Palsy (1, 4, 5), Autism Spectrum Disorder (6), Duchenne Muscular Dystrophy (7, 8), and many other conditions (9–14). Accelerometer measurements show potential in infant populations as a tool to predict later acquisition of neurodevelopmental and neuromuscular disorders (15–17). Across the pediatric literature, studies are highly variable with respect to the number of sensors used, sensor placement, wearing time, and how data extracted from the sensors are analyzed. Many studies derive conceptually similar variables (e.g., relative symmetry between the limbs, magnitude of accelerations), but use different calculation methods. The varied protocols and variable calculations, in addition to small sample sizes, are barriers to the comparison of data across papers and across populations.

Pediatric researchers need information about upper limb accelerometry variables in typically-developing children. The availability of normative or referent data would facilitate analyses and interpretation of data from various diagnostic groups, especially rare disease populations (10). Availability of such data could also reduce the burden placed on research teams; researchers will not need a “control” sample to interpret the data from their target clinical population. Methodology used to produce the referent data could potentially provide a standardized way to collect and analyze accelerometry data, and enhance comparisons across papers in the future.

The purpose of this report was to provide referent accelerometry data on 25 variables in typically-developing children, ages 3-17 years. We utilized a harmonized data set from three previous cohorts, which all collected data from both wrists using similar protocols. Upper limb accelerometry data, as an adjunct to established assessment methods, may be of high value for two key reasons. First, it can measure motor behavior on ratio scales, which do not require different forms for different ages. And second, compared to lower limb accelerometry, upper limb methodologies can be used consistently throughout infancy and into adulthood, both as walking is acquired and as degenerative diseases progress and limit walking in later life.

## Methods

This is a secondary analysis using harmonized data from typically-developing children from three previous studies (6, 18, 19). Each of these studies employed a similar protocol (20) to collect bilateral wrist accelerometry from pediatric participants. Inclusion criteria for this analysis were: (1) age between 3 and 17 yrs; (2) data from caregiver-reported medical history, questionnaires, and in-person assessments indicated the participant was typically-developing; and (3) accelerometer recordings had >10 h of data during waking hours. To confirm participants were typically-developing, study team members triangulated available data from: parent-reported medical history and questionnaires (all 3 cohorts), medical record information (partially available in 2 cohorts; used most often to confirm exclusion from this typically-developing analysis by a neurodevelopmental diagnosis), and in-person developmental assessments (2 cohorts) in motor, cognitive, language, intellectual, and social-communication domains. Standardized questionnaires and assessments for each cohort were as follows. Konrad et al. 2022 (6) used the Developmental Coordination Disorder Questionnaire [DCDQ (21), or the Little DCDQ (22) for younger children], the Connor-3 Parents' Rating Scale [(23) or the Connors-Early Childhood Scale for younger children (24)], and the Social Responsiveness Scale-2 [SRS-2 (25, 26), or the SRS-2 Preschool for younger children (27)]. Konrad et al. 2024 (19) used all of the above along with the Movement Assessment Battery for Children-2 [MABC-2 (28)] and the Kaufman Brief Intelligence Test [KBIT-2 (29)]. Hoyt et al. 2019 (18) used the Ages and Stages Questionnaire [(30) or the MABC-2 or MABC-2 Checklist for older children] and the Child Behavior Checklist (31). Caregivers provided written informed consent and participants 6 yrs and up provided their verbal assent for the originating studies, which permitted de-identified data to be shared and used for subsequent analyses.

The wrist-worn devices used were either Actigraph GT3X-BT or GT9X-Link Activity Monitors (Actigraph, Pensacola FL). These devices have 3-axis, solid-state accelerometers with a dynamic range of ±8 gravitational units. The devices were worn on both upper limbs, just above the radial and ulnar styloid processes. Bands to attach the devices varied based on caregiver-preference and size of the wrists. Participants were asked to wear the devices for two days (6, 19) during waking hours or four, non-consecutive days (18) for 24 h. The wearing periods were decided during the design of each contributing study. In general, the decision was a compromise between obtaining sufficient data and the willingness of the family and child to wear the sensors. For two of the cohorts (6, 19), the decision to limit the wearing period to two days was because a portion of the clinical population of interest were children with Autism Spectrum Disorder (their data not included here), who often have sensory sensitivities. The devices could be removed for water activities or if the participant was experiencing skin irritation. Devices were returned to the study team after the wearing period with shipping materials provided to the caregiver, dropped off by caregiver or family-member, or picked up at the participant's home.

### Accelerometry data processing and variables extracted

Here we provide a brief overview of the data processing, with more extensive details provided within each original study (6, 18, 19). Data were downloaded from devices using ActiLife 5 or 6 software (Actigraph, Pensacola FL), plotted, and visually-inspected to confirm wearing time. This secondary analysis used the extracted 30 Hz data and down-sampled 1 Hz data. The 30 Hz data were bandpass filtered from 0.2–12 Hz to remove constant linear acceleration components, such as gravity and non-human accelerations (e.g., riding in a car or elevator). Likewise, the 1 Hz data were bandpass filtered between 0.25–2.5 Hz and converted to activity counts (1 activity count = 0.001664 g), using ActiLife's proprietary process. The accelerometer data from each axis were transformed into vector magnitudes by taking the square root of (*x*^2^ + *y*^2^ + *z*^2^). Accelerometer variables were then calculated from the time-series, vector magnitude values.

Data from all three cohorts were reprocessed to ensure all variables were computed the same way across cohorts. Table 1 provides the variables and their conceptual definitions, while Supplementary Table S1 provides the formulae in annotated R code. Variables were calculated and categorized according to four characteristics of human movement: duration, intensity, symmetry, and complexity as previously described (6, 32, 33). When multiple ways to compute a construct or variable were available in the literature, we defaulted to the mathematically simpler option, e.g., calculated use ratio as a measure of symmetry vs. mono-arm use index (18). We also selected the calculations where the values are not dependent on the length of the recording period. For examples, average jerk is calculated instead of cumulative jerk (7), and average acceleration magnitude is calculated instead of cumulative or total magnitude (6). Other variables were selected because preliminary data suggest they may differentiate between and/or be predictive of future neurodevelopmental diagnosis, e.g., variance of the frequency spectrum (6, 16). Duration, intensity, symmetry, and some complexity variables were computed from the 1 Hz time-series data. Other complexity variables required higher time resolution so the 30 Hz time series data was used (see Table 1). Variables were computed from the entire recording period, except for entropy of the dominant and non-dominant limb, which were computed from the hour of maximum activity (6). Periods of sleep were not removed due to their trivial effect on upper limb accelerometry variables [Miller et al. 2024 (www.researchsquare.com/article/rs-3838376/v1)]. For variables reflecting the intensity and variance in activity counts, reported values were converted back to gravitational units (gs), since this unit of measure is device-independent. The factor that was used was 1 activity count = 0.001664 gs, which is an approximation when used as a reconversion factor [for more information see (34, 35)]. While research into the psychometric properties and clinical utility of these variables is at various stages of scientific development (36), we report on 25 variables to provide a comprehensive set of variables from which others may select the most appropriate for their research and clinical efforts.

**Table 1:** Variables extracted from the time series accelerometry data.

Movement Characteristic	Variable	Definition
Duration (hrs)	Total movement time	Total time that either limb is moving.^a^
	Time	Time the limb is moving.^a^
	*[D & ND]*	
	Isolated time	Time that one limb is moving and the other limb is still.^a^
	*[D & ND]*	
	Simultaneous time	Time that both limbs are moving.^a^
Intensity (gs^b^)	Magnitude	The median acceleration magnitude when the limb was moving, quantifying how intense the limb movement tends to be.
	*[D & ND]*	
	Bilateral Magnitude	The sum of the median acceleration magnitude from both limbs. An aggregate measure of overall upper limb movement intensity.
	Peak Magnitude	The maximum acceleration magnitude that occurred during the recording period.
	*[D & ND]*	
Symmetry	Use ratio^c^	Ratio of the duration of non-dominant to dominant limb movement.
	Magnitude ratio^c^	Ratio of the magnitude of non-dominant to dominant limb movement.
	Variation ratio^c^	Ratio of the variance of non-dominant to dominant limb movement.
	Jerk asymmetry index	Ratio of the average jerk magnitude between the limbs as (jerk_non−dom_—jerk_dom_)/jerk_non−dom_ + jerk_dom_). An index of 0 = similar smoothness in the movement of the limbs. Values are bounded between −1 and +1.
Complexity	Variance	The standard deviation of the acceleration magnitude when the limb was moving, quantifying how variable the movement tends to be. Units = gs.
	*[D & ND]*	
	Entropy	The time series variability of the accelerations from the limb during the maximum hour of activity. Calculated as sample entropy. Higher values indicate a more random signal.
	*[D & ND]*	
	Jerk^d^	Average rate of change of acceleration in gravitational units across the recording period, when the limb was moving. Quantifies the smoothness of the limb movements where lower values indicate smoother movement. Units = g/s.
	*[D & ND]*	
	Mean of Frequency Spectrum^d^	The weighted mean of the component frequencies from the acceleration time series. An aggregate measure of the frequency at which the participant tends to move their upper limb, which may relate to disordered movement. Units = hz.
	*[D & ND]*	
	Variance of Frequency Spectrum^d^	The weighted standard deviation of the component frequencies from the acceleration time series. Quantifies the degree to which the frequency of the participant's upper limb movement fluctuates. Higher values may indicate more complex movement patterns (16). Units = hz
	*[D & ND]*	

D, dominant side; ND, non-dominant side.

^a^ Each second of data is binned as movement vs. no movement based on the threshold of 2 activity counts. Seconds of movement over the recording period are summed and then divided by 3,600 to convert to hours.

^b^ Converted from device-specific activity counts to gravitational units (g = m/s^2^), (1) Actigraph activity count is approximately equal to 0.001664 gs when processed as described in this paper.

^c^ Use, magnitude, and variation ratios equal 1 when the limbs are active for the same amount of time, magnitude, and variability, respectively.

^d^ Calculated from the 30 Hz time-series data.

### Statistical analyses

All statistical analyses were performed in the R environment version 4.3.1. Descriptive statistics and distribution plots were generated for each variable. Relationships between variables on the dominant and non-dominant limbs and the influence of age were determined using Pearson Product Moment correlations. Sex differences for each variable were evaluated using t tests. We next examined the stability of the variables 3 ways. The first way examined the absolute agreement between measurements using Intraclass Correlation Coefficients (ICCs) with an ICC[2,k] model (37). The second way we examined the magnitude of the day-to-day variation was with a mixed-effect model. Each sensor variable was estimated by: $y_{ij}=X\beta +\gamma_{i}+\epsilon_{ij}$, where ***β*** included fixed effects of Age (linear, quadratic, and cubic effects), Sex (male v. female) and Day (linear effect) and a random intercept for each subject. *γ*_i_. The standard deviation of the residuals, $\sigma_{\epsilon_{ij}}$, thus represents unexplained day to day variation, *j*'s, in a given variable, after systematic sources of variance have been removed. The third way expressed the relative magnitude of the day-to-day variation, computing a coefficient of variation (SD/mean) for each participant and then taking the mean of these values. Last, we explored relationships between variables, also using Pearson Product Moment correlations. For these correlations, observations across days were averaged to obtain one observation for each participant. Each of these analyses resulted in a large number of statistical tests with substantial opportunity for spurious findings. Thus, we restricted statistical significance *within* each analysis by the number of variables analyzed using Bonferroni corrections, resulting in an adjusted significance threshold of *p* < 0.002 (0.05/25).

Towards the goal of making these data useful to others in pediatric research, we also built a web-based display of the data and the resulting analyses via an R Shiny object (38). This provided a web-based, graphical user interface for users to view to suit their own needs. This interface uses the same source data and shows many of the analyses presented here.

## Results

**Figure 1:**
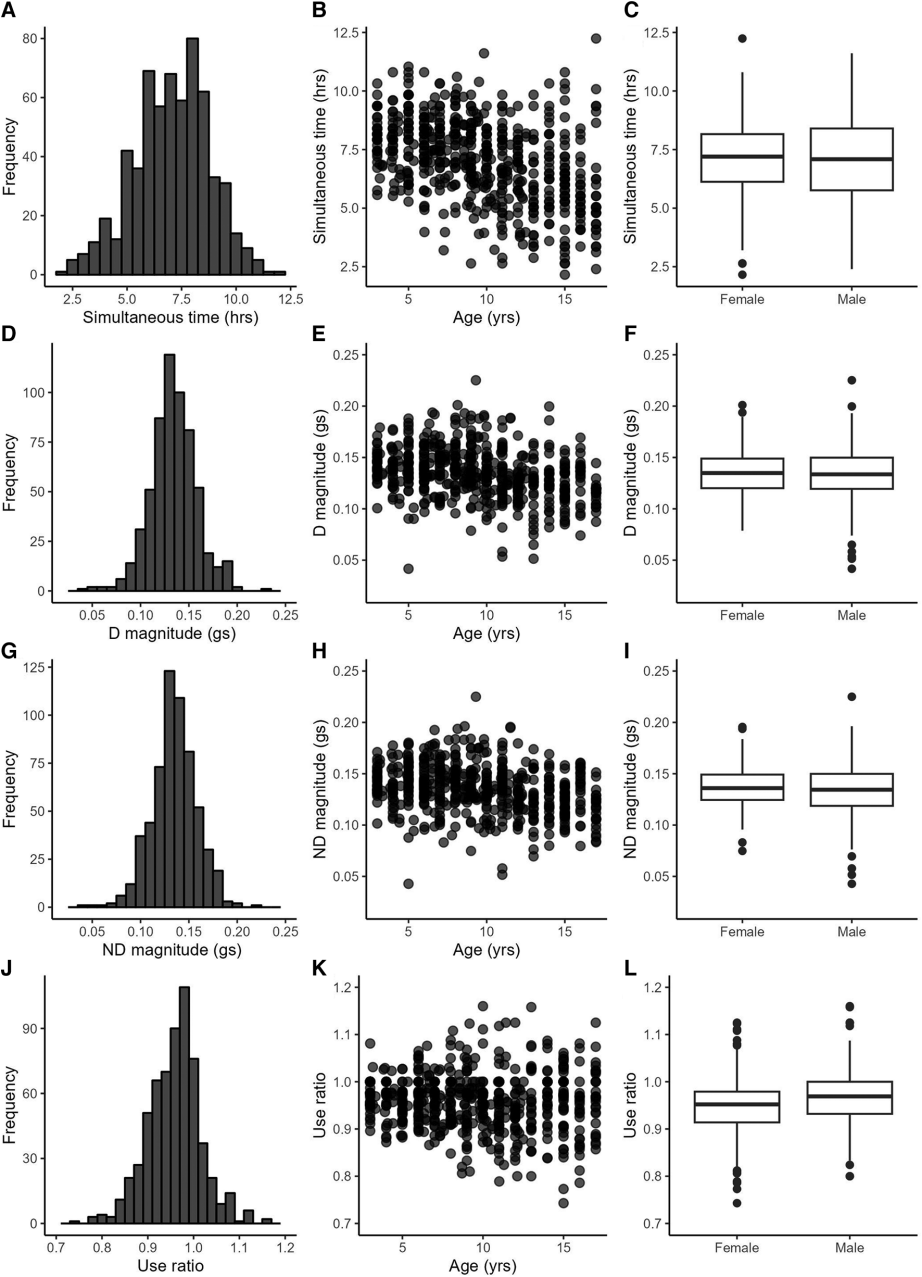
Example variables from the duration (**A**–**C**), intensity (**D**–**I**) and symmetry (**J**–**L**) categories. Left column: variable distributions; middle column: scatterplots with age; right column: boxplots by sex.

**Figure 2:**
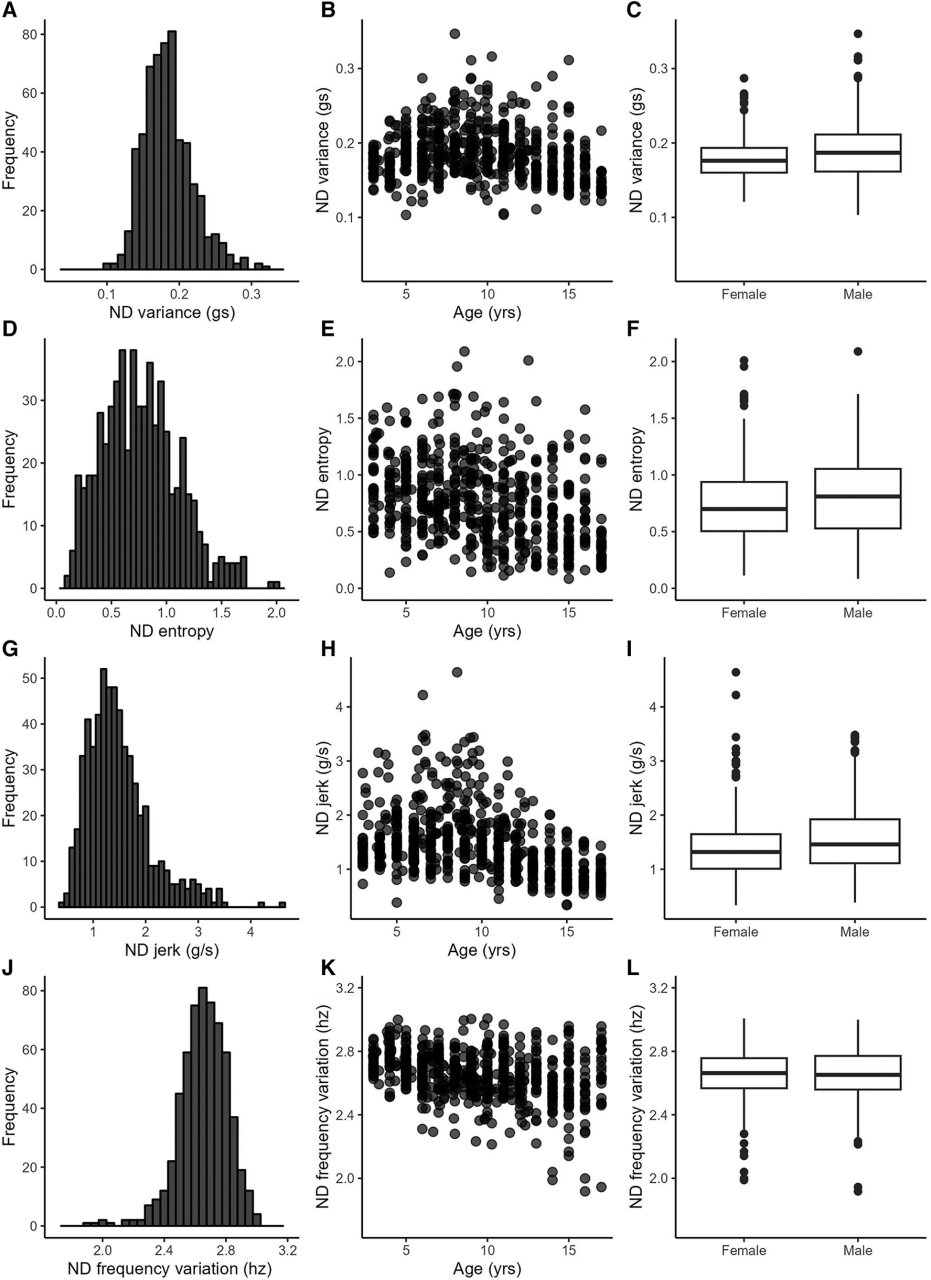
Example variables from the complexity category. Left column: variable distributions; middle column: scatterplots with age; right column: boxplots by sex.

The included data set consisted of 622 recording days from 222 typically-developing individuals collected between 2012 and 2022. Data from multiple days were grouped by individual for the analyses, but are displayed by recording day in figures. Participant demographics are provided in the top of Table 2. Sex was equally distributed, and the sample contained mostly white, non-Hispanic, socioeconomically-advantaged participants [as quantified by the Area Deprivation Index (39)]. The number of participants and the number of recording days for each age are provided in Supplementary Table S2. Descriptive statistics from the 25 accelerometry variables are provided in the bottom of Table 2. Figures 1, 2 show distributions, scatterplots with age, and mean values by sex for 8 example variables. Most, but not all variables were normally distributed, as can be seen in the left columns of Figures 1, 2 and by the similarities between the mean and 2nd quartile (median) values in Table 2.

**Table 2:** Demographic characteristics of the sample (top) and descriptive statistics for the accelerometry variables (bottom).

	Descriptive statistics						Missing data	
Age (yrs)	9.4 ± 3.8 (3–17)						0	
Hand dominance	94% Right, 6% Left						3%^a^	
Sex	52% Male, 48% Female						0	
Ethnicity	94% Non-Hispanic/Latinx, 6% Hispanic/Latinx						3%	
Race	90% White, 4% Black or African American, 3% Asian, 3% More than one race						3%	
Area Deprivation Index^b^	36 ± 21 (5–93)						3%	
Device wear time/day (hrs)	21.4 ± 4.8 (10–25)						0%	
	Mean	SD	SE	Min	1st quartile	2nd quartile	3rd quartile	Max
Duration variables (hrs)								
Total movement time	10.55	1.65	0.07	5.76	9.56	10.80	11.76	15.84
D time	8.93	1.65	0.07	4.08	7.92	9.11	10.08	12.80
ND time	8.56	1.62	0.07	4.08	7.44	8.71	9.71	12.65
D isolated time	1.79	0.51	0.02	0	1.44	1.68	2.16	3.84
ND isolated time	1.44	0.43	0.02	0	1.20	1.44	1.68	3.6
Simultaneous time	7.05	1.74	0.07	2.16	6.00	7.2	8.24	12.24
Intensity variables (gs)								
D magnitude	0.134	0.024	0.001	0.042	0.120	0.134	0.149	0.225
ND magnitude	0.135	0.023	0.001	0.043	0.122	0.135	0.149	0.225
Bilateral magnitude	0.270	0.046	0.002	0.084	0.242	0.270	0.298	0.450
D peak magnitude	1.952	0.359	0.014	0.833	1.727	1.982	2.189	3.082
ND peak magnitude	1.887	0.348	0.014	0.762	1.666	1.923	2.129	3.059
Symmetry variables								
Use ratio	0.96	0.06	0.002	0.74	0.92	0.96	1.00	1.16
Magnitude ratio	1.01	0.09	0.004	0.72	0.96	1.00	1.05	1.36
Variation ratio	0.99	0.06	0.002	0.73	0.96	0.99	1.03	1.22
Jerk asymmetry index	−0.018	0.048	0.002	−0.371	−0.046	−0.018	0.008	0.172
Complexity variables								
D variance (gs)	0.187	0.036	0.001	0.098	0.162	0.182	0.209	0.318
ND variance (gs)	0.185	0.035	0.001	0.103	0.161	0.181	0.204	0.347
D entropy	0.82	0.34	0.01	0.10	0.56	0.80	1.03	2.04
ND entropy	0.77	0.35	0.01	0.08	0.52	0.74	0.98	2.09
D jerk (g/s)	1.55	0.61	0.02	0.35	1.11	1.45	1.84	4.40
ND jerk (g/s)	1.48	0.61	0.02	0.34	1.07	1.37	1.75	4.64
D mean frequency (hz)	3.42	0.35	0.01	2.10	3.19	3.42	3.65	4.66
ND mean frequency (hz)	3.42	0.30	0.01	2.56	3.22	3.42	3.62	4.38
D frequency variance (hz)	2.68	0.16	0.006	1.90	2.59	2.68	2.798	3.21
ND frequency variance (hz)	2.65	0.16	0.006	1.92	2.56	2.66	2.76	3.01

D, dominant side; ND, non-dominant side.

^a^ When hand dominance was missing, the participant was assumed to be right handed for data processing purposes.

^b^ Area Deprivation Index national percentiles, based on US Census block group. Values range from 0 to 100, with higher values indicating greater socioeconomic deprivation (39).

Table 3 provides statistics from the side-to-side, age, and sex relationships, ICCs, and day-to-day variance. Values from the dominant and non-dominant limb were highly correlated with each other. Figures 1D,G illustrate this for the dominant and non-dominant magnitudes. The side-to-side similarities are also mathematically apparent in the symmetry variables, with an example of the use ratio shown in Figure 1J. Many of duration, intensity, and complexity variables declined gently (e.g., Figures 1B,E,H, 2E,H), while the symmetry variables were stable across the 3–17 yr. age range (e.g., Figure 1K). Sex differences were found in only three variables: the dominant limb peak magnitude and variance, and the variation ratio. ICC values ranged from 0.66–0.94. The values of the sensors variables fluctuated day-to-day. Typical day-to-day variance, as quantified by the standard deviation of the mixed effect model residuals, provides the fluctuations in units of each variable. The coefficient of variation across days presents the day-to-day fluctuations as a percentage, to allow readers to consider the relative stability of each variable.

**Table 3:** Statistics from analyses on side-to-side relationships, age, sex, and day-to-day stability.

	Corr. between D & ND sides	Corr. with age	Sex differences^a^	ICC[2,k]	Typical day-to-day variation^b^	Coefficient of variation across days^c^
Total movement time	–	***−0***.***26***	0.23	***0***.***82***	1.14	8.6%
D time	***0***.***95***	***−0***.***38***	0.28	***0***.***82***	1.14	10%
ND time	*** ***	***−0***.***40***	0.14	***0***.***84***	1.08	10%
D isolated time	***0***.***48***	***0***.***34***	0.10	***0***.***76***	0.37	17.1%
ND isolated time	*** ***	***0***.***35***	−0.04	***0***.***73***	0.33	18%
Simultaneous time	***–***	***−0***.***44***	0.19	***0***.***85***	1.11	12.8%
D magnitude	***0***.***89***	***−0***.***32***	0.001	***0***.***85***	0.017	9.5%
ND magnitude	*** ***	***−0***.***30***	0.002	***0***.***85***	0.015	9.4%
Bilateral magnitude	***–***	***−0***.***32***	0.005	***0***.***85***	0.030	9.3%
D peak magnitude	***0***.***72***	−0.11	***−0***.***187***	***0***.***79***	0.256	10.3%
ND peak magnitude	*** ***	−0.14	−0.101	***0***.***77***	0.257	10.8%
Use ratio	***–***	−0.12	−0.01	***0***.***76***	0.04	3.7%
Magnitude ratio	***–***	0.15	0.007	***0***.***85***	0.06	4.4%
Variation ratio	***–***	0.06	***0***.***026***	***0***.***74***	0.05	3.8%
Jerk asymmetry index	***–***	−0.06	−0.007	***0***.***86***	0.032	138%^d^
D variance	***0***.***94***	−0.18	***−0***.***013***	***0***.***82***	0.025	10.2%
ND variance	*** ***	−0.20	−0.008	***0***.***82***	0.024	9.9%
D entropy	***0***.***89***	***−0***.***39***	−0.03	***0***.***66***	0.28	29.2%
ND entropy	*** ***	***−0***.***39***	−0.07	***0***.***69***	0.29	31.8%
D jerk	***0***.***97***	***−0***.***39***	−0.21	***0***.***93***	0.31	17.7%
ND jerk	*** ***	***−0***.***38***	−0.19	***0***.***94***	0.29	17.6%
D mean frequency	***0***.***86***	***−0***.***26***	0.10	***0***.***82***	0.24	5.5%
ND mean frequency	*** ***	***−0***.***36***	0.07	***0***.***86***	0.19	4.6%
D frequency variance	***0***.***85***	***−0***.***33***	0.002	***0***.***71***	0.13	3.7%
ND frequency variance		***−0***.***41***	−0.003	***0***.***79***	0.11	3.4%

Statistical significance (*p* < 0.002) marked by bold italics for correlations and comparisons.

^a^ For sex differences, negative values indicate higher values in males. Values are in units of the variable.

^b^ Typical day-to-day variation in units of the variable, estimated from the residuals of the mixed effect model.

^c^ Coefficient of variation shows the relative day-to-day variation (mean value of $CoV_{i}=\;s_{i}/{\bar{x}}_{i}$ for each participant, *i*, across the sample).

^d^ Jerk asymmetry index mean values approach zero, mathematically inflating the daily variation when expressed relative to the mean.

**Figure 3:**
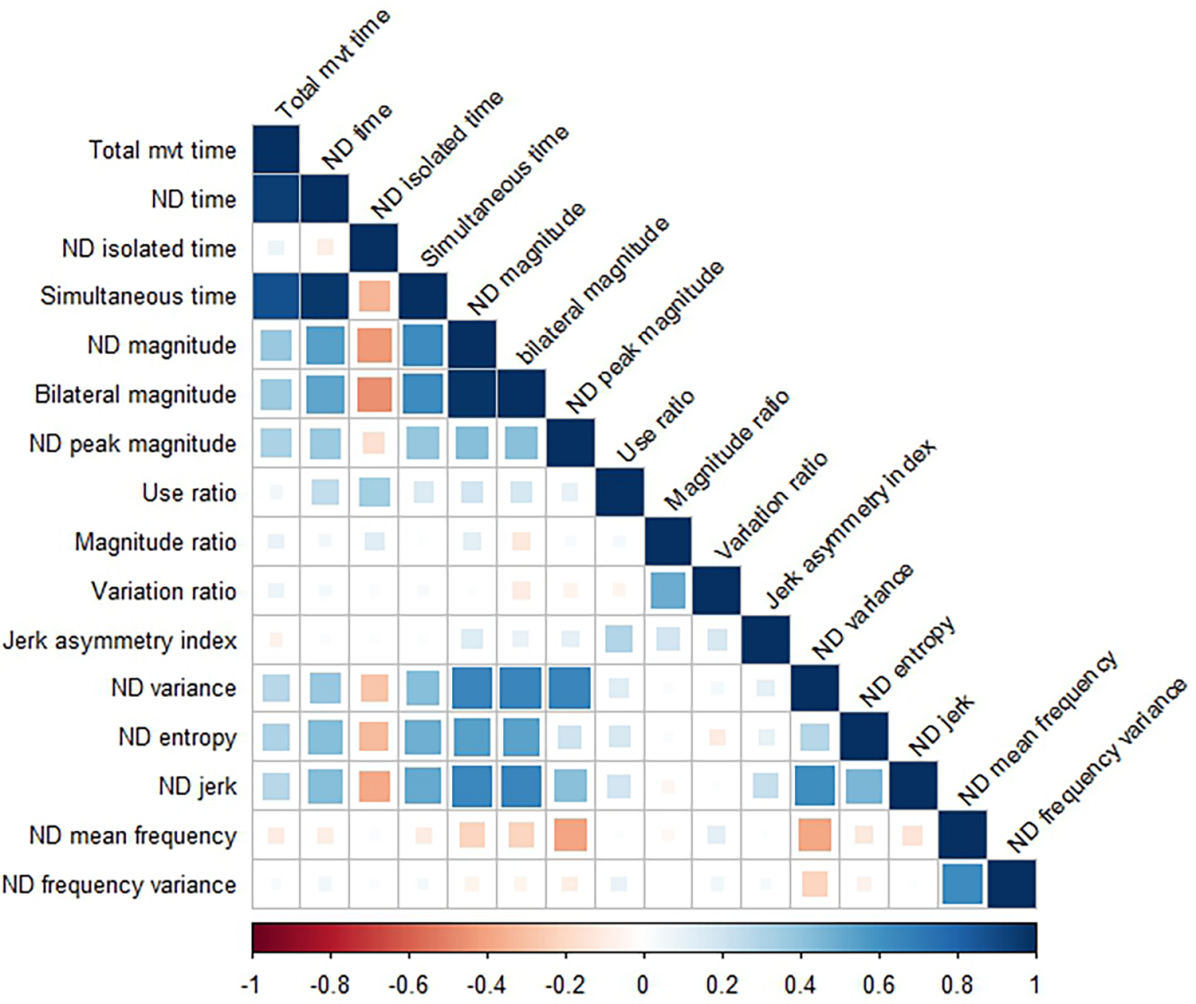
Correlation matrix showing relationships between variables on the non-dominant (ND) side. The correlation matrix for the dominant side (not shown) is nearly identical. Correlation coefficients > ±0.20 were statistically significant, with values weaker than that represented by empty or nearly empty squares in the matrix.

Figure 3 shows the correlation matrix between variables for the non-dominant side. Correlation coefficient values were nearly identical within the dominant side, so only one correlation matrix is shown here. Correlations stronger than *r* = 0.20 (positive or negative) were statistically significant at the corrected *p* value of 0.002. Duration and intensity variables were moderately to strongly related to each other such that longer durations were associated with more intense movements. The exception to this is isolated time, such that longer durations of single limb only movement where associated with less intense movement. Duration and magnitude variables were also related to several complexity variables, such that longer and more intense movement was associated with more variable (ND variance), less uniform (ND entropy), and less smooth (ND jerk) movement.

The analyses for each variable can be viewed with via the R Shiny Object at https://langlab.shinyapps.io/harmonized_data/. The left-side menu allows the user to select the sensor variable category, and then the specific variables, age ranges, and sex of interest. User selections determine which data populate the table at the top and the related graphs. As one example, a user could select the sensor variable category of “Intensity”, the sensor variable of “ND_magnitude”, the age from 5 to 5.99, and the sex to “Both” to view the values and graphs specific to 5 yrs olds. As another example, one could set the age from 12 to 14 and select only “Males” to see values for middle school–aged boys for this same or other variables. Two notes for these data are: (1) as age ranges become more narrow and the sample sizes decrease, the computed correlations vary widely; and (2) plots and statistics in the visualization are based on all available observations, not aggregated to one observation per subject. As data used for these analyses become publically available, the links on the bottom of the left-side menu will be updated.

## Discussion

This report provides comprehensive information on 25 variables extracted from bilateral, wrist-worn accelerometers from typically developing children, ages 3 to 17 years. The majority of variables were similar on dominant and non-dominant sides, moderately-associated with age, and not different between boys and girls. ICC values were moderate to excellent. Variation within individuals across days ranged from 3% to 32% in the 25 variables, excluding the jerk asymmetry index. These data provide a quantification of many of the constructs of interest found in the literature [e.g., relative symmetry between the two limbs (4, 11, 18, 40) quantified by four variables here], and generate the calculations so that values are not dependent on the length of the recording period [e.g., average jerk instead of cumulative jerk (7), average acceleration magnitude instead of total (6)] or the device used [values here in gravitational units and not device-specific activity counts (1)]. Employing uniform calculations will facilitate comparison of data across papers and populations. Where it is possible to make direct comparisons with other literature, our values indicate that upper limb movement is less symmetrical and less intense in samples of children with cerebral palsy, perinatal stroke, or brachial plexus injury (1, 11, 40, 41). Similarly, estimated comparisons to children with Duchenne Muscular Dystrophy suggest that increasing Brooks scale ratings would correspond with intensity estimates that fall further and further below the mean values presented here, with a rating of 1 (least severe disease state) corresponding to −1 SD here, and a rating of 4 (most severe disease state) corresponding to >4 SDs below our mean (8). With the use of wearable movement sensors rapidly increasing, these data provide key, referent information for researchers as they design studies, and analyze and interpret data from neurodevelopmental and other pediatric clinical populations.

Variables recorded from the dominant and non-dominant sides were strikingly similar and highly correlated with each other. These data mirror referent accelerometry data from adults (42), despite the universal phenomenon of hand dominance (43). While one might think of the dominant limb being more active in daily life, these data confirm that most upper limb activity involves the use of both sides, e.g., one hand being used to grasp a container and the other hand being used to open the container. Note that these data do not imply that the limbs are doing the same movements at the same time; the accelerometry variables cannot identify the specific movements that were made. Instead, the data imply that characteristics of limb movements, as quantified by these variables over many hours, are highly similar between the dominant and non-dominant sides. Thus for future studies, wearing one sensor on either limb may be sufficient when a research team is studying pediatric conditions that affect movement on both sides. Wearing sensors on both sides may remain important for conditions that have an asymmetrical effect on movement (4, 5, 40, 44).

Three other results have important implications for the use of wearable motion sensors in pediatric populations. *First*, most variables decreased slightly with increasing age and had high variability within each age (e.g., scatterplot panels in Figures 1,2). These findings are consistent with the general decrease in physical activity in children as they move into adolescence (45). Thus, researchers can select a wide range around their target ages, when identifying referent control or comparison data for their population of interest. *Second*, unlike with physical activity measures (45), sex had small influences on only three of 25 variables. Indeed the between sex difference for each variable (middle column of Table 3) was much smaller than the day-to-day variability (adjacent, right column of Table 3) and minimal compared to the sample standard deviation (2nd column of Table 2). Researchers therefore can feel confident in using referent data from both sexes for most variables. *And third*, the estimates of day-to-day variance (right two columns in Table 3) indicate that researchers may want to consider a trade-off between stability of the variable of interest and participant burden. These estimates are computed from 2 to 4 wearing days, which is much shorter than the weeks to months data collection used for determining physical activity (45). The moderate to excellent ICC values indicate that averaging across the 2–4 days produces stable values. For variables with lower day-to-day variance (e.g., use ratio, D and ND frequency variables), sensor wearing periods of 2 days may be sufficient to capture a precise estimate. For variables with higher day-to-day variance (e.g., D and ND jerk and entropy), longer sensor wearing periods may be needed to achieve greater precision. Many clinical studies often require that assessments occur repeatedly over weeks or months. In our experience, participants tend to be more compliant with repeated wearing when the individual wearing periods are shorter. The values in Tables 2, 3 can also assist the design of longitudinal studies, providing data to estimate the amount of change that may be detectable (or considered real change) over time for each variable. The R Shiny object serves as an important extension of this paper, giving anyone the opportunity to explore these data for their own research or clinical questions.

Two main limitations of this data set are its demographic make-up and its sample size. As with many existing data sets, persons of color and under-resourced children were under-represented. While the sample included 622 recording days, the data came from 222 children. We intentionally labeled it “referent” data, since the sample was not large enough to merit the label of “normative” data across the age range of 3–17. An additional limitation to ponder is that values were not always calculated from the same duration of wearing time. We used 10 h of recording as the minimum included here, i.e., the minimum to sufficiently sample movement activity for a day. Average device wearing time was high at 21.4 h (Table 2), with a median value of 24 h. Different recording durations could have the largest influence on the Duration variables, since variables in other categories are primarily statistical features of the signal and not sums. Most of the included recording days that were <24 h were in the younger children, where parents helped the child don the devices after waking up and then removed them at bedtime. Despite the shorter recording time in some participants, we were able to detect similar relationships between age and the Duration variables as with age and the Intensity and Complexity variables. These relationships suggest that the influence of different recording lengths may be minimal.

## Conclusions

We have provided comprehensive referent data on 25 variables extracted from wrist-worn motion sensors in typically-developing children between the ages of 3 and 17. These data can serve as comparator values for ongoing and future studies in clinical pediatric populations, and may be of particular high value for rare or orphan diseases (10). Further data collection for children from 6 months to 3 years of age is underway as part of NIH R01MH723123 and data sets from 6 adult cohorts are being reprocessed with the same methods. As new cohorts from our group and others are studied using a similar protocol, the field will benefit from adding these data, especially data from under-represented communities, to this harmonized database.

## Data Availability

The raw data supporting the conclusions of this article will be made available by the authors, without undue reservation.
