# Corrigendum: Referent data for investigations of upper limb accelerometry: harmonized data from three cohorts of typically-developing children

**DOI:** 10.3389/fped.2024.1406314

**Published:** 2024-05-17

**Authors:** Catherine E. Lang, Catherine R. Hoyt, Jeffrey D. Konrad, Kayla R. Bell, Natasha Marrus, Marghuretta D. Bland, Keith R. Lohse, Allison E. Miller

**Affiliations:** ^1^ Program in Physical Therapy, Washington University School of Medicine, St. Louis, MO, United States; ^2^ Program in Occupational Therapy, Washington University School of Medicine, St. Louis, MO, United States; ^3^ Department of Neurology, Washington University School of Medicine, St. Louis, MO, United States; ^4^ Department of Psychiatry, Washington University School of Medicine, St. Louis, MO, United States

**Keywords:** wearable sensors, upper limb, movement, accelerometers, pediatrics, behavior, outcomes

A Corrigendum on Referent data for investigations of upper limb accelerometry: harmonized data from three cohorts of typically-developing children By Lang CE, Hoyt CR, Konrad JD, Bell KR, Marrus N, Bland MD, Lohse KR and Miller AE. (2024) Front. Pediatr. 12:1361757. doi: 10.3389/fped.2024.1361757

In the published article, there was an error. The reverse conversion factor by which activity counts were converted to gravitational units is presented as a precise, mathematical constant. In the authors' discussions with the Actigraph Corporation science team, it is best to consider this conversion factor as an approximation. To explicitly convey this two new references have been added.

A correction has been made to **Methods**, *Accelerometry data processing and variables extracted*, 2nd paragraph. The corrected paragraph and two new references appear below.

“Data from all three cohorts were reprocessed to ensure all variables were computed the same way across cohorts. Table 1 provides the variables and their conceptual definitions, while Supplementary Table S1 provides the formulae in annotated *R* code. Variables were calculated and categorized according to four characteristics of human movement: duration, intensity, symmetry, and complexity as previously described (6, 32, 33). When multiple ways to compute a construct or variable were available in the literature, we defaulted to the mathematically simpler option, e.g., calculated use ratio as a measure of symmetry vs. mono-arm use index (18). We also selected the calculations where the values are not dependent on the length of the recording period. For examples, average jerk is calculated instead of cumulative jerk (7), and average acceleration magnitude is calculated instead of cumulative or total magnitude (6). Other variables were selected because preliminary data suggest they may differentiate between and/or be predictive of future neurodevelopmental diagnosis, e.g., variance of the frequency spectrum (6, 16). Duration, intensity, symmetry, and some complexity variables were computed from the 1 Hz time-series data. Other complexity variables required higher time resolution so the 30 Hz time series data was used (see Table 1). Variables were computed from the entire recording period, except for entropy of the dominant and non-dominant limb, which were computed from the hour of maximum activity (6). Periods of sleep were not removed due to their trivial effect on upper limb accelerometry variables [Miller et al. 2024 (www.researchsquare.com/article/rs-3838376/v1)]. For variables reflecting the intensity and variance in activity counts, reported values were converted back to gravitational units (gs), since this unit of measure is device-independent. The factor that was used was 1 activity count = 0.001664 gs, which is an approximation when used as a reconversion factor [for more information see (34, 35)]. While research into the psychometric properties and clinical utility of these variables is at various stages of scientific development (34), we report on 25 variables to provide a comprehensive set of variables from which others may select the most appropriate for their research and clinical efforts.”

34. Neishabouri A, Nguyen J, Samuelsson J, Guthrie T, Biggs M, Wyatt J, et al. Quantification of acceleration as activity counts in ActiGraph wearable. *Sci Rep*. (2022) 12(1):11958. doi: 10.1038/s41598-022-16003-x

35. Actigraph. What are counts? (2018). Available online at: https://actigraphcorp.my.site.com/support/s/article/What-are-counts (Accessed December 10, 2018).

The authors apologize for this error and state that this does not change the scientific conclusions of the article in any way. The original article has been updated.

In the published article, there was an error in the footnote for Table 1 (superscript b) as published. The reconversion factor should have been expressed as an approximation. The corrected footnote (superscript b) appears below.

^b^Converted from device-specific activity counts to gravitational units (g = m/s^2^), (1) Actigraph activity count is approximately equal to 0.001664 gs when processed as described in this paper.

The authors apologize for this error and state that this does not change the scientific conclusions of the article in any way. The original article has been updated.

