# Supplementary figures and images for: Referent data for investigations of upper limb accelerometry: harmonized data from three cohorts of typically-developing children
Source: Front Pediatr. 2024 Mar 1;12:1361757. doi: 10.3389/fped.2024.1361757 (PMC10940427; doi:10.3389/fped.2024.1361757)

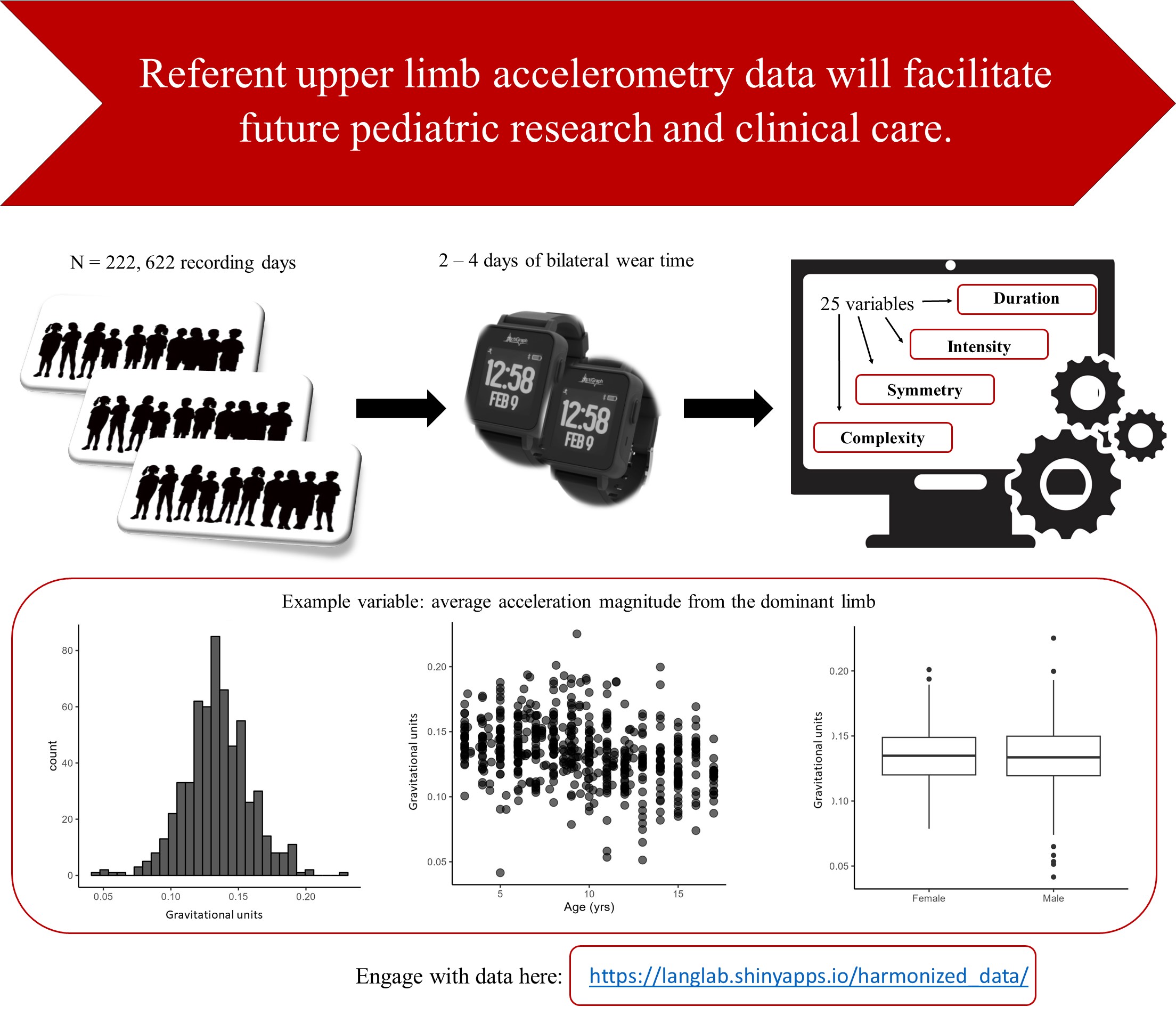

Supplement: Supplementary file 1 [file Image1.jpeg]
