# Supplementary material for: Referent data for investigations of upper limb accelerometry: harmonized data from three cohorts of typically-developing children
Source: Front Pediatr. 2024 Mar 1;12:1361757. doi: 10.3389/fped.2024.1361757 (PMC10940427; doi:10.3389/fped.2024.1361757)
Supplement: Supplementary file 2 [file Table1.docx]

**Supplemental Table 1.** Formulae in R code for each variable. Green font, preceded by #, indicates comments in the R code. The code calculates values for the left and ride side, and then later values are assigned to dominant and non-dominant labels accordingly.

| Duration variables | Calculated from the 1 Hz vector magnitude time series data, with variables named below as LVMData and RVMData.  Threshold for classifying as movement was set at 2 Actigraph activity counts  (or 0.003328 gravitational units, after filtering to remove the effects of gravity)  Calculated duration values are converted from seconds to hours and rounded to 2 decimal points. |
| --- | --- |
| Total movement time | #Find the frames where one or the other limb was moving.  Mvt <- which(LVMData >= Threshold \| RVMData >= Threshold)  #Count up the movement frames  TotalMovementTime <- round((length(Mvt)/3600), 2) |
| Time | #Find the frames where the limb was moving.  LCount <- which(LVMData >= Threshold)  RCount <- which(RVMData >= Threshold)  #Count up the movement frames.  LeftTime <- round((length(LCount)/3600), 2)  RightTime <- round((length(RCount)/3600), 2) |
| Isolated time | #Find the frames where one limb is moving and the other is not.  TempLData <- which(LVMData >= Threshold & RVMData < Threshold)  TempRData <- which(LVMData < Threshold & RVMData >= Threshold)  #Count up the isolated movement frames.  IsolatedLeftTime <- round((length(TempLData)/3600), 2)  IsolatedRightTime <- round((length(TempRData)/3600), 2) |
| Simultaneous time | #Find the frames where both limbs are moving.  TempData <- which(LVMData >= Threshold & RVMData >= Threshold)  #Count up the simultaneous movement frames.  SimultaneousTime <- round((length(TempData)/3600), 2) |
| Intensity variables | Intensity values were calculated from activity counts, converted to gravitational units, and rounded to 3 decimal points. |
| Magnitude | #Median value of the frames in which the limb is moving.  LeftMagnitude <- round((median(LVMData[LCount]) * 0.001664), 3)  RightMagnitude <- round((median(RVMData[RCount]) * 0.001664), 3) |
| Bilateral Magnitude | #Sum the left and right values.  BilateralMagnitude <- LeftMagnitude + RightMagnitude |
| Peak Magnitude | #Find the peak values.  LeftPeakMagnitude <- round(max(LVMData[LCount]) * 0.001664), 3)  RightPeakMagnitude <- round(max(RVMData[RCount]) * 0.001664), 3) |
| Symmetry variables | Left and right values are assigned to the appropriate dominant and non-dominant labels. Ratio variables are rounded to 3 decimal points. |
| Use ratio | UseRatio <- round(NondominantTime/DominantTime, 3) |
| Magnitude ratio | MagnitudeRatio <- round(NondominantMagnitude/DominantMagnitude, 3) |
| Variation ratio | VariationRatio <- round(NondominantVariance/DominantVariance, 3) |
| Jerk asymmetry index | JerkAsymmetryIndex <- (NondominantJerk - DominantJerk) / (NondominantJerk + DominantJerk) |
| Complexity variables |  |
| Variance | #From the 1 Hz time series data.  #Standard deviation of the acceleration magnitudes when the limb was moving.  #Calculated in activity counts, then converted to gravitational units and rounded.  LeftVariance <- round((sd(LVMData[LCount]) * 0.001664), 3)  RightVariance <- round((sd(RVMData[RCount]) * 0.001664), 3) |
| Entropy | #From the 1 Hz time series data.  #Calculated from the most active hour on the most active side in the recording period.  #Calculated using the R sample_entropy function and rounded to 3 decimal places.  RightEntropy <- round(sample_entropy(RVM_MaxHour, edim = 2, 0.2*sd(RVM_MaxHour), tau = 1) ,3)  LeftEntropy <- round(sample_entropy(LVM_MaxHour, edim = 2, 0.2*sd(LVM_MaxHour), tau = 1) ,3) |
| Jerk | #From the 30 Hz time series data, same naming conventions for the L and R..  #Calculate time-series jerk data, the time derivative of acceleration.  TP <- 1/30 #the sampling interval  #Left  LJerk <- vector()  Ljerk[1] <- 0  I <- 1  for (i in 1:(nrow(LVMData) - 1)) {  A <- LVMData[i+1, ycol]  B <- LVMData[i, ycol]  LJerk[i] <- (A – B ) / TP  }  #Right  RJerk <- vector()  RJerk[1] <- 0  I <- 1  for (i in 1:(nrow(RVMData) - 1)) {  A <- RVMData[i+1, ycol]  B <- RVMData[i, ycol]  RJerk[i] <- (A – B ) / TP  }  #Remove the frames where the jerk was 0  NoJerkL <- which(LJerk == 0)  LJerk <- LJerk[-NoJerkL]  NoJerkR <- which(RJerk == 0)  RJerk <- RJerk[-NoJerkR]  #Find mean of absolute values and round to 3 decimal points  LeftJerk <- round(mean(abs(LJerk)), 3)  RightJerk <- round(mean(abs(RJerk)), 3) |
| Mean of Frequency Spectrum | #From the 30 Hz time series data.  #Find the frequency spectrum, kernal filter = 5  SpectrumLVM <- spectrum(LVMData, log='no', span=5, plot = F)  SpectrumRVM <- spectrum(RVMData, log='no', span=5, plot = F)  #Divide spectrum by sampling interval to create per time instead of per sampling interval  LFreq <- SpectrumLVM$freq/0.033333  RFreq <- SpectrumRVM$freq/0.033333  #Multiply the spectral density by 2 so that the area under the periodogram  #equals the variance of the time series.  LDensity <- 2*SpectrumLVM$spec  RDensity <- 2*SpectrumRVM$spec  #Calculate weighted means of frequency spectrum and round to 3 decimal points.  MeanLeftFreq <- round(weighted.mean(LFreq, LDensity), 3)  MeanRightFreq <- round(weighted.mean(RFreq, RDensity), 3) |
| Variance of Frequency Spectrum | #From the 30 Hz time series data.  #Weighted variance function from  #https://stat.ethz.ch/pipermail/r-help/2008-July/168762.html  weighted.var <- function(x, w, na.rm = FALSE) {  if (na.rm) {  w <- w[i <- !is.na(x)]  x <- x[i]  }  sum.w <- sum(w)  sum.w2 <- sum(w^2)  mean.w <- sum(x * w) / sum(w)  (sum.w / (sum.w^2 - sum.w2)) * sum(w * (x - mean.w)^2, na.rm =  na.rm)  }  #Following code above, find weighted variance of the frequencies present.  LWeightedVariance <- weighted.var(LFreq, LDensity)  RWeightedVariance <- weighted.var(RFreq, RDensity)  #Find the standard deviation and round to 3 decimal points.  LeftFreqVariance <- round(sqrt(LWeightedVariance), 3)  RightFreqVariance <- round(sqrt(RWeightedVariance), 3) |

.

**Supplemental Table 2.** Number of participants and recording days for each age.

| **Age (yrs)** | **Number of participants** | **Number of recording days** |
| --- | --- | --- |
| 3 | 15 | 45 |
| 4 | 13 | 35 |
| 5 | 11 | 40 |
| 6 | 25 | 62 |
| 7 | 19 | 48 |
| 8 | 20 | 51 |
| 9 | 24 | 55 |
| 10 | 16 | 41 |
| 11 | 16 | 47 |
| 12 | 18 | 45 |
| 13 | 8 | 25 |
| 14 | 9 | 30 |
| 15 | 12 | 44 |
| 16 | 8 | 26 |
| 17 | 8 | 28 |
| Total | 222 | 622 |
